# Promutagenicity of 8-Chloroguanine, A Major Inflammation-Induced Halogenated DNA Lesion

**DOI:** 10.3390/molecules24193507

**Published:** 2019-09-27

**Authors:** Yi Kou, Myong-Chul Koag, Seongmin Lee

**Affiliations:** The Division of Chemical Biology and Medicinal Chemistry, College of Pharmacy, The University of Texas at Austin, 2409 University Avenue, Austin, TX 78712, USA; yik109@hotmail.com (Y.K.); mckoag@gmail.com (M.-C.K.)

**Keywords:** 8-chloro-2′-deoxyguanosine, G to C transversion mutations, DNA polymerase, translesion synthesis, mismatch, X-ray crystallography

## Abstract

Chronic inflammation is closely associated with cancer development. One possible mechanism for inflammation-induced carcinogenesis is DNA damage caused by reactive halogen species, such as hypochlorous acid, which is released by myeloperoxidase to kill pathogens. Hypochlorous acid can attack genomic DNA to produce 8-chloro-2′-deoxyguanosine (ClG) as a major lesion. It has been postulated that ClG promotes mutagenic replication using its *syn* conformer; yet, the structural basis for ClG-induced mutagenesis is unknown. We obtained crystal structures and kinetics data for nucleotide incorporation past a templating ClG using human DNA polymerase β (polβ) as a model enzyme for high-fidelity DNA polymerases. The structures showed that ClG formed base pairs with incoming dCTP and dGTP using its *anti* and *syn* conformers, respectively. Kinetic studies showed that polβ incorporated dGTP only 15-fold less efficiently than dCTP, suggesting that replication across ClG is promutagenic. Two hydrogen bonds between *syn*-ClG and *anti*-dGTP and a water-mediated hydrogen bond appeared to facilitate mutagenic replication opposite the major halogenated guanine lesion. These results suggest that ClG in DNA promotes G to C transversion mutations by forming Hoogsteen base pairing between *syn*-ClG and *anti*-G during DNA synthesis.

## 1. Introduction

DNA bases are subjected to halogenation [1]. Exogenous chlorine and its related hypochlorite compounds widely exist in modern society as disinfectants, bleaches, and industrial reagents [2,3,4]. Additionally, there have been many reports revealing that direct contact with even the diluted form of these compounds can introduce mutation [5,6,7,8]. More importantly, hypochlorite compounds can be produced endogenously [9,10]. During inflammation and infection, various cells in the host immune system are activated [11,12]. The NADPH oxidase generated in this process efficiently produces H_2_O_2_. Catalyzed by myeloperoxidase (MPO), H_2_O_2_ reacts with chloride ion to generate hypochlorous acid HOCl [10,13,14]. Another enzyme capable of such peroxidation reaction of halide ions is eosinophil peroxidase (EPO) [14,15]. An important discovery of HOCl is that it can easily perform transhalogenation reactions using nucleobases at the physiological level [10,16,17,18]. This is significant in that: (1) It suggests the underlying reason for bactericidal effect of the MPO or EPO in the host defense system: reactive halogen species mutate DNA bases to kill the pathogens [9,19,20]; (2) additionally, it provides one reasonable explanation for inflammation-derived carcinogenesis, which occupies 20% of all cancer types [21], and many of the organ-specific carcinogenesis have been shown to have a cause-and-effect relationship attributed to local inflammation, such as liver and gastric cancer, resulting from viral and bacterial infections [22,23,24,25]. It is possible that extra HOCl generated during chronic or severe inflammation can modify various DNA bases that elicit the mutation, and finally develop into cancer [26].

HOCl and other halogenating reagents possess the ability to damage DNA of various bases [27], forming 5-halo-dC (hereafter ClC) and 8-halo-dG and -dA lesions. 5-chloro-2′-deoxy cytosine from inflammation-mediated DNA damage model studies has been reported to cause genomic instability: the chlorination brings both steric obstruction to interfere with the affinities of DNA binding enzymes [28] and electronegativity, weakening the H-bonds between the ClC:G base pair [29]. What is worse is that the ClC can perform fraudulent methylation on unmethylated CpG dinucleotide, whose reverse process by 5-aza-deoxycytidine indicates interference on the epigenetic level [30]. The 5-chloro-uracil, a derivative product of ClC, showed enhanced miscoding probability [31] with the ClU:G mismatch revealing pH-dependent structural change [29]. The reduction potential of Guanine makes it the most easily oxidized and damaged base in DNA [32,33]. By measuring the melting temperature, the steric bulk of C8 halogen atom destabilizes dG:dC base pairs by destabilizing the *anti*-conformation of dG: the greater the atomic radius or bond length, the less stable the analogue in a base pair opposite dC [34]. It has been reported that nicotine can enhance the formation of 8-chloro-dG (hereafter ClG). In addition, taurine, the most abundant free amino acid in leukocyte cytosol, can greatly enhance the formation of promutagenic 8-bromo-dG (BrG) [35,36]. Many other studies confirm that the ClG-mediated tobacco inflammation can develop into carcinogenesis [25]. ClG, the predominant halogenated lesion [16], has shown its promutagenicity to delete and miscode probabilities with DNA polymerase kappa and other polymerases [26].

**Figure 1:**
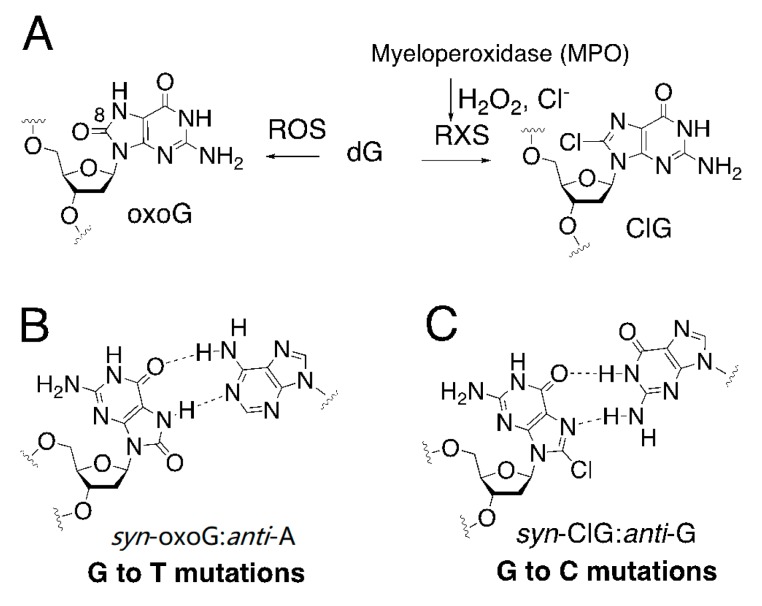
Promutagenicity of oxidative DNA lesions. (**A**) Generation of oxidized purines by reactive oxygen species (ROS) and reactive halogen species (RXS). (**B**) Mutagenic base pairing between 8-oxoguanine and adenine, which involves two hydrogen bonds between a *syn* conformer of 8-oxo-dG and an *anti*-conformer of dA. This mutagenic base pair can cause G to T transversion mutations. (**C**) Potential mutagenic base pairing between a *syn* conformer of 8-chloro-dG and an *anti*-conformer of dG, which can lead to G to C transversion mutations.

To explore the potential mutagenicity of ClG in a detailed mechanism approach, we use human DNA polymerase β (polβ) as a model enzyme for DNA polymerases that undergo an open-to-closed conformational change during correct nucleotide insertion: It is much less likely that polβ encounters ClG lesion at a templating position. Instead, Y-family translesion synthesis DNA polymerases or replicative DNA polymerases are more likely to catalyze across ClG lesions at physiologically relevant conditions. Polβ, an X-family DNA polymerase, fills a short-nucleotide gap that is formed in base excision repair pathway as well as synthesizes opposite undamaged DNA. The protein contains an N-terminal lyase domain (8 kDa) and a C-terminal polymerase domain (31 kDa). The polymerase domain can be further divided into thumb, fingers, and palm subdomains, typically observed in DNA polymerases [37]. Polβ, the smallest mammalian DNA polymerase lacking 3′ to 5′ proofreading exonuclease activity, has an active site that is highly sensitive to the presence of mismatches [38,39]. ClG could be promutagenic when bypassed by polβ. While the N7-H of oxoG can act as a H-bond donor to form a Hoogsteen base pair with incoming dATP [40,41], the C8 chlorination of guanine can make an N7 base pair with incoming dGTP via its Hoogsteen edge (Figure 1A–C), thereby promoting G to C transversion mutations.

To study the translesion synthesis of ClG in the template strand by polβ, we report herein three crystal structures of polβ in complex with DNA containing templating ClG. These polβ-ClG structures provide insights into promutagenic replication across ClG. We also report steady-state insertion kinetics of polβ incorporating dNTP opposite ClG, which shows that templating ClG increases misincorporation efficiency compared to dG.

## 2. Results and Discussion

### 2.1. Steady-State Kinetic Studies

Using the steady-state kinetic method, we determined kinetic parameters for nucleotide incorporation opposite ClG by polβ (Table 1). In the presence of Mg^2+^, nucleotide insertion efficiency of dCTP opposite ClG was ~60-fold (0.55 vs. 34.54) lower than that of dCTP opposite normal dG. The use of Mn^2+^ increased the dCTP insertion by 23-fold (0.55 vs. 12.61), showing that the insertion efficiency of dCTP opposite ClG was greatly influenced by the types of metal cofactors. The insertion efficiency of dGTP opposite ClG was only ~15-fold (0.55 vs. 0.038) lower than that of dCTP opposite ClG, highlighting the promutagenicty of templating ClG. Substituting Mn^2+^ for Mg^2+^ increased dGTP insertion by ~32-fold (0.038 vs. 1.15). Considering all the dNTPs conditions as listed here, this suggests that ClG:G mismatched base pair frequently occurs, when compared to other mismatched ClG base pairs.

### 2.2. Structure of A Single-Nucleotide Gapped Binary Complex of Polβ with Templating ClG

**Figure 2:**
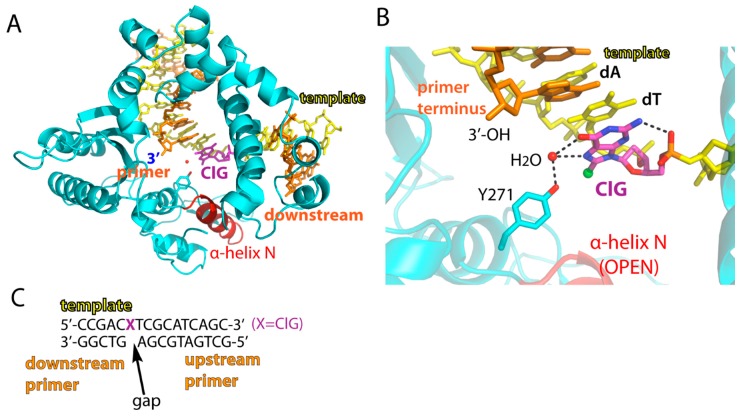
Structure of a single-nucleotide gapped binary complex of polβ and ClG-containing DNA. (**A**) Overall structure of polβ in complex with templating ClG. Templating ClG is shown as colored magenta. The minor groove-interacting α-helix N is colored red. (**B**) Active-site view of the polβ:ClG binary complex. Hydrogen bonds are indicated in dotted lines. Note a water-mediated hydrogen bond network between Tyr271 and ClG and stabilization of *syn* ClG via a hydrogen bond between N2 of ClG and the 5′ phosphate backbone oxygen. Protein is an open conformation. (**C**) DNA sequence used for crystallization of a single-nucleotide gapped polβ complex. The 5′ of the downstream primer is phosphorylated.

To determine conformation of ClG in a templating position, we solved crystal structure of polβ bound to DNA containing a single nucleotide gap opposite templating ClG. The structure of the gapped binary complex of polβ:ClG, refined to 2.2 Å resolution, showed an open protein conformation, where the minor groove recognition residues Asn279 and Arg283 on the α-helix N were ~10 Å away from templating ClG (Figure 2 and Table 2). The unpaired ClG in a templating position preferentially adopted a *syn* conformation (Figure 2B). The *syn* conformation of ClG was stabilized by the water-mediated hydrogen bonds with O6 and N7 from its Hoogsteen edge and the OH from the side chain of Tyr271. ClG also fixed itself by an intramolecular hydrogen bond between N2 of ClG and 5′-phosphate oxygen (Figure 2B).

### 2.3. Ternary Structure of Polβ with Templating ClG Paired with an Incoming dCTP Analog

**Figure 3:**
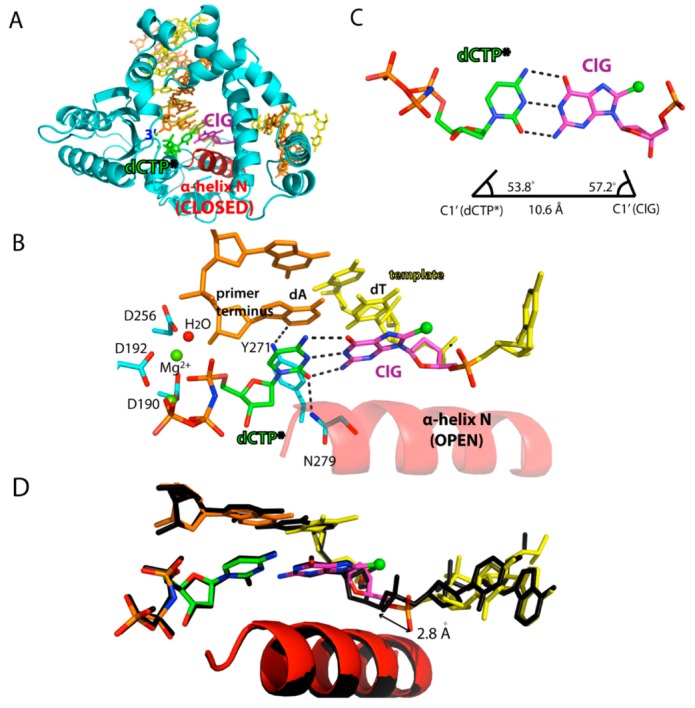
Ternary structure of polβ incorporating non-hydrolyzable dCTP analog opposite templating ClG. (**A**) Overall view of the polβ-ClG:dCTP* ternary complex structure. ClG is shown in magenta and the incoming dCTP* is colored green. The α-helix N, which is shown in red, forms a closed conformation. (**B**) Active-site view of the polβ-ClG:dCTP* ternary complex structure. The templating ClG is an *anti*-conformation and forms three Watson-Crick hydrogen bonds with incoming dCTP*. The minor groove edges of the incoming dCTP and primer terminus are recognized by Asn279 and Tyr271, respectively. Hydrogen bonds are indicated in dashed lines. Metal cofactors are shown in green spheres and water is shown in red sphere. (**C**) Base pair geometry of dCTP*:ClG in the active site of polβ. (**D**) Superposition of the polβ-ClG:dCTP* structure (multicolor) with the polβ-dA:dUTP* ternary complex structure (black). The distance between the 5′-phosphate oxygen of the templating bases is shown.

As shown in the kinetics results, with the incoming dCTP, polβ showed slow catalysis opposite the templating ClG. To gain insights into this, we obtained ternary structures of polβ in precatalytic complex with an incoming nonhydrolyzable dCMPNPP (hereafter dCTP*) to be paired with templating ClG with both Mg^2+^ metal ions (Figure 3). The use of this nucleotide analog has been suggested to retain the same structure as dCTP [42,43,44]. This precatalytic ClG:dCTP*(Mg^2+^) ternary structure was refined to 2.0 Å (Figure 3A and Table 2).

When templating ClG was base paired with dCTP*, the protein underwent an open-to-closed conformational change to sandwich the nascent base pair between the primer terminus base pair and α-helix N (Figure 3A,B). In the polβ-ClG:dCTP* ternary structure, templating G was in *anti*-conformation instead of *syn* (Figure 3B). Templating *anti*-ClG formed three hydrogen bonds with dCTP*, with its base pair geometry being essentially identical to that of dG:dCTP (Figure 3C). The Watson-Crick base pair of ClG:dCTP* caused the conformational change from *syn*-ClG to *anti*-ClG. The change of *syn* to *anti*-conformation by itself would definitely change the H bondings of ClG in the base pair as well as in the surrounding active site. By comparing the binary and ternary dCTP* structures, the conformational change from *syn* to *anti* caused four H bonds broken and more than six new H bonds were formed. Additionally, coordination improved significantly, indicating that the base pairing with dCTP* for *anti*-conformer of ClG was thermodynamically more stable than its gapped *syn* conformer. The metal ion in the ClG:dCTP*-Mg^2+^ ternary structure was a near perfect octahedral coordination geometry for both nucleotide binding ion and catalytic ion. Additionally, the minor groove recognition residues, Asn 279 and Arg 283, stabilized the dCTP*:ClG base pair by making minor groove H-bond contacts. The upstream primer terminus was further stabilized by hydrogen bond contact with Tyr 271. However, despite the correct Watson-Crick base pair of ClG:dCTP*, our kinetics indicated that insertion efficiency for the ClG with incoming dCTP is significantly lower (~50X) than that of normal dG (Table 1). Our structure provided a perspective: when ClG was at the templating position, the 3′-OH of the upstream primer strand was not in an expected in-line position for nucleophilic attack of the α phosphate from incoming dCTP*. Instead, from alignment to a normal G:C structure, the 3′ primer terminus sugar rotated about 37° back (C2′ (ClG)-C3′-C2′ (dG)), making an ineffective 3′-OH position for the precatalytic state towards the α-phosphate of incoming dCTP*. Furthermore, the primer terminus 3′-OH was 4.5 Å away from the Pα of the phosphate, which was about 1 Å longer than the 3′-OH-Pα distance observed in the polβ-dG:dCTP ternary structure. Thus, although incoming dCTP* can still make an ideal Watson-Crick base pair with the template ClG, the α-helix N takes the closed conformation, the nucleotide and the catalytic binding metal ions are nearly ideally coordinated, and the poor coordination of upstream primer 3′-OH makes the nucleotidyl transfer reaction not favorable as compared to the dG:dCTP structure. The innate drive might come from the long-distance interaction that originated from the shift caused by ClG: The presence of the C8 chlorine might cause a potential steric clash with the oxygen on the 5′-phosphate, which can shift the template strand and induce subsequent structural reorganization of the phosphate backbone. The non-optimal position of the primer terminus 3′-OH for the S_N_2 backside attack and phosphate backbone reorganization near the templating ClG could slow the insertion of dCTP opposite the templating ClG.

### 2.4. Ternary Structure of Polβ with Templating ClG Paired with an Incoming dGTP Analog

**Figure 4:**
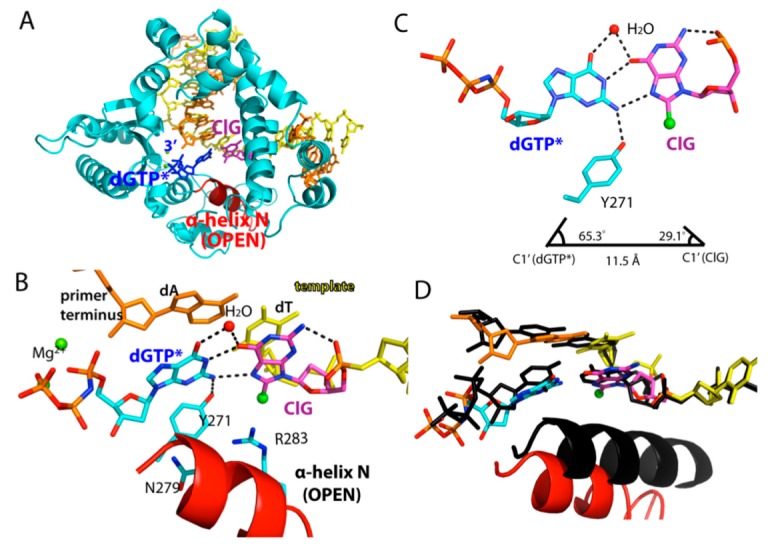
Ternary structure of polβ in complex with ClG:dGTP* base pair. (**A**) Overall view of polβ-ClG:dGTP* ternary complex structure. ClG is in purple; incoming dGTP* in green. α-Helix N takes a semi-open conformation. (**B**) Active-site view of the polβ-ClG:dGTP* structure. The α-helix N is in open conformation. Asn279 and Arg283 do not make minor groove contacts. Hydrogen bonds are indicated in dotted lines. (**C**) Base pair geometry of ClG and dGTP*. Note that the ClG takes a *syn* conformation, forming H-bonds with incoming dGTP* via its Hoogsteen edge and water mediation. (**D**) Superposition of the polβ-ClG:dGTP* ternary complex structure (multicolor) with the polβ-dA:dUTP* ternary complex structure (black).

To gain structural insights into mutagenic replication across ClG, we solved a ternary complex structure of polβ incorporating dGTP* opposite the templating ClG, which was refined to 2.4 Å (Figure 4 and Table 2).

The base pair conformation of the polβ-ClG:dGTP* ternary complex was different from those of the published polβ structures with mismatches (e.g., G:T). Published polβ mismatched structures, except for the 8-oxoguanine, typically show a staggered base pair conformation. On the contrary, ClG and dGTP* formed a coplanar base pair conformation. The ClG was in a *syn* conformation and created Hoogsteen hydrogen bonds with incoming dGTP* (Figure 4B,C). O6 and N7 of ClG were engaged in hydrogen bonds with N1 and N2 of dGTP*, respectively. A water-mediated hydrogen bond between O6 of ClG and O6 of dGTP* further stabilized the ClG:dGTP base pair. The observation of coplanar ClG:dGTP base pair conformation suggests that the promutagenic base pair is relatively well accommodated in the replication site. The polβ structure with coplanar ClG:dGTP conformation is consistent with only 15-fold decrease in catalytic efficiency for the ClG:dGTP insertion, compared to that for the ClG:dCTP insertion.

While the ClG:dGTP* base pair formed a coplanar conformation, the protein adopted an open conformation, where the α-helix N was ~10 Å away from the nascent ClG:dGTP* base pair. The nucleotide’s minor-groove edge contacts with amino acid residues were shown to promote incorporation of correct nucleotide in the replication site. In particular, polβ uses Tyr271, Asn279, and Arg283 to sense the minor groove edges of the primer terminus base, incoming nucleotide, and templating base, respectively (PDB ID 3LK9). Tyr271, which engaged in minor groove interaction with primer terminus base in the ClG:dCTP* complex, was hydrogen bonded to N2 of dGTP*. In addition, Asn279 and Arg283 did not engage in hydrogen bonds with the minor groove edges.

These hydrogen bonds stabilized the Hoogsteen *syn*-ClG:*anti*-G base pair, making it a coplanar conformation, despite the fact that the C1′-C1′ distance of the base pair was elongated to 11.5Å, and λ angles deviated to 65° and 29°, respectively (Figure 4C). The formation of a catalytically less competent protein conformation for the ClG:dGTP* base pair suggests that the enzyme is sensitive to the presence of non-Watson-Crick base pair geometry and deters misincorporation by adopting open conformation.

Our studies provide the structural basis for the dual coding potential of major inflammation-induced halogenated DNA lesions [45,46,47,48,49,50]. The single nucleotide gapped structure of the polβ-ClG complex shows that, in the absence of an incoming nucleotide, templating ClG preferentially exists as a *syn* conformer. The steric clash between chlorine and C5′, and the repulsive interaction between chlorine and O4′, makes *syn*-ClG conformation more favorable than *anti*-ClG conformation. In the presence of incoming dCTP, ClG adopts an *anti*-conformation and forms three Watson-Crick hydrogen bonds with dCTP. On the other hand, in the presence of incoming dGTP, ClG adopts a *syn* conformation and forms two Hoogsteen hydrogen bonds with *anti*-dGTP. The base pairing between ClG and dGTP is further stabilized by water-mediated hydrogen bonds that connect the two O6 atoms of ClG and dGTP. Crystal structures show that both *syn*-ClG:*anti*-dG and *anti*-ClG:*anti*-dG base pairs are readily accommodated in the catalytic site of a DNA polymerase, suggesting the dual coding potential of ClG.

Comparison of structures of polβ in complex with ClG- and 8-oxoguanine (8-oxoG)-containing DNA explains why 8-oxoG is more promutagenic than ClG. Although both 8-oxoG and ClG can use their Hoogsteen edges to base pair with incoming dATP and dGTP, respectively, 8-oxoG:dATP has a more favorable base pair geometry than ClG:dGTP in the replication site. In particular, λ angles for the C1′ (8-oxoG) and C1′ (dATP) of 8-oxoG:dATP are ~55°, which is essentially identical to that for the normal Watson-Crick base pair. On the contrary, λ angles for ClG:dGTP are 29° and 65°, which significantly deviates from the normal Watson-Crick base pair geometry.

Our study is consistent with the observation that nucleotidyl transfer across ClG by high fidelity DNA polymerase is slower, yet preferentially produces ClG:G mismatches. The dual coding potential of ClG could lead to G to C transversion mutations, which could contribute to inflammation-induced cancer development. Currently, no known DNA glycosylases have shown to cleave ClG, suggesting that the persistence of ClG in DNA could facilitate mutagenic replication.

## 3. Materials and Methods

### 3.1. Synthesis of ClG (8-chloro-dG) Phosphoramidite

The synthesis of ClG phosphoramidite followed published procedures [34]. Briefly, after the dehydration of deoxyguanosine monohydrate by pyridine coevaporation, dG was treated with isobutyryl chloride in pyridine to give 3′,5′,N2-isobutyrylated dG. The protected dG was then chlorinated at the C8 position by *N*-chlorosuccinimide (NCS) in tetrahydrofuran at 25 °C. After removal of the 3′ and 5′ isobutryl groups by methanolic sodium methoxide, the resulting compound was 5′-tritylated and 3′-phosphitylated to yield 8-CldG phosphoramidite, which was used for solid phase DNA synthesis.

### 3.2. DNA Sequences

Oligonucleotides were purchased from Integrated DNA Technologies (IDT, Coralville, IA, USA). ClG-containing oligonucleotides were custom-synthesized by Midland Certified Reagent Co. (Midland, TX, USA). Oligonucleotides were purified by the manufacturer, and their sequences were confirmed by MALDI-TOF mass spectrometry. DNA used for crystallographic studies consisted of a 16-mer template, a complementary 10-mer primer, and 5-mer downstream oligonucleotides. The template DNA sequence used for crystallization was 5′-CCGAC(ClG)TCGCATCAGC-3′. The upstream primer sequence was 5′-GCTGATGCGA-3′. The downstream oligonucleotide sequence was 5′-pGTCGG-3′, where 5′ terminus was phosphorylated. The DNA sequence almost identical to a published ternary complex structure (PDB ID 1BPY) was used to minimize sequence-dependent structural differences. The oligonucleotides were mixed and annealed to give a 1 mM mixture of gapped DNA, as described [38].

### 3.3. Steady-State Kinetics

Steady-state kinetic parameters for dNTP (dCTP, dGTP, dATP, dTTP) insertion opposite templating ClG by polβ (1–5 nM) were determined using the same procedure, as described previously [38]. Each oligonucleotide was annealed in a hybridization buffer (10 mM Tris-HCl, pH 7.5, and 1 mM EDTA) to prepare a substrate for polβ containing a single-nucleotide gap opposite guanine or ClG. Polymerase activities were determined using a reaction mixture containing 50 mM Tris-HCl, pH 7.5, 100 mM KCl, 5 mM MgCl_2_ or MnCl_2_, 80 nM single-nucleotide gapped DNA, and varying concentrations of incoming dNTP. The nucleotidyl transfer reactions were initiated by the addition of the enzyme and stopped with a gel-loading buffer containing 95% formamide with 20 mM EDTA, 45 mM Tris-borate, 0.1% bromophenol blue, and 0.1% xylene cyanol. The quenched samples were separated on 18% or 20% urea polyacrylamide gels. The gels were analyzed using a PhosphorImager (Molecular Dynamics, Chicago, IL, USA) to quantify product formation. The *k*_cat_ and *K*_m_ were determined by fitting the reaction rates at various dNTP concentrations to the Michaelis-Menten equation. Each experiment was repeated at least three times to measure the average of the kinetic results. The efficiency of nucleotide insertion was calculated as *k*_cat_/*K*_m_. The relative frequency of dNTP incorporation opposite the templating ClG was determined as *f* = (*k*_cat_/*K*_m_)[dNTP:ClG]/(*k*_cat_/*K*_m_)[dCTP:dG] [51].

### 3.4. Protein Expression and Purification, Protein-DNA Co-crystallization

Polβ was expressed and purified from *Escherichia coli* with minor modifications of the method described previously [38]. The binary polβ complex containing templating ClG in a single-nucleotide gapped DNA was prepared under conditions similar to those described previously [42]. Polβ was complexed with a single-nucleotide gapped DNA containing a 16-mer template (5′-CCGAC(ClG)GCGCATCAGC-3′), a complementary 10-mer primer (5′-GCTGATGCGC-3′), and a 5-mer downstream oligonucleotide (5′-pGTCGG-3′). The resulting polβ-DNA complex was used to obtain binary and ternary complex crystals in the absence or presence of an incoming nucleotide, respectively. The ternary polβ-DNA complex co-crystals with nonhydrolyzable dNTP analogs paired with templating ClG in a single-nucleotide gap at the active site were grown in a solution containing 50 mM imidazole, pH 7.5, 14–23% PEG3400, and 350 mM sodium acetate as described previously [42]. Crystals were cryo-protected in mother liquor supplemented with 12% ethylene glycol and were flash-frozen in liquid nitrogen.

### 3.5. Data Collection and Refinement

Diffraction data were collected at 100 K using either a Rigaku MicroMax-007 HF microfocus x-ray generator (Rigaku, Woodlands, TX, USA) with R-Axis IV++ imaging plate area detector or the beamline 5.0.3 Advanced Light Source at Berkeley Center for Structural Biology (Berkeley, CA, USA). All diffraction data were processed using HKL 2000 (HKL Research, Charlottesville, VA, USA) [52]. The structures of the binary polβ complex with templating ClG in a single-nucleotide gapped DNA and the ternary complex of polβ with templating ClG paired with dNTP analog were solved by molecular replacement with polβ with a single-nucleotide gapped DNA (PDB code 1BPX) as the search model. The model was built using COOT and refined using PHENIX software [53,54]. MolProbity was used to make Ramachandran plots [55].

## Figures and Tables

**Table 1:** Kinetic parameters of dNTPs insertion opposite the ClG lesion.

Template:dNTP (Metal Ion)	*K*_m_ (μM)	*k*_cat_ (10^−3^ s^−1^)	*k*_cat_/*K*_m_ (10^−3^ s^−1^·μM^−1^)	*f* ^a^
dG:dCTP (Mg^2+^)	0.59 ± 0.03	20.38 ± 0.50	34.54	1
ClG:dCTP (Mg^2+^)	6.52 ± 0.81	3.59 ± 0.23	0.55	1.6 × 10^−2^
ClG:dGTP (Mg^2+^)	22.96 ± 0.23	0.87 ± 0.05	0.038	1.1 × 10^−3^
ClG:dCTP (Mn^2+^)	1.31 ± 0.22	16.52 ± 1.01	12.61	3.7 × 10^−1^
ClG:dGTP (Mn2+)	3.92 ± 0.09	4.52 ± 0.34	1.15	3.3 × 10^−2^

^a^ Relative efficiency: (*k*_cat_/*K*_m_)[dNTP:ClG]/(*k*_cat_/*K*_m_)[dCTP:dG].

**Table 2:** Data collection and refinement statistics.

PDB Code	ClG-Gapped Binary 6CLY	ClG:C-Mg^2+^ Ternary 6CPQ	ClG:G-Mg^2+^ Ternary 6CRH
Space Group	P2_1_	P2_1_	P2_1_
Cell constants			
a (Å) b c α (°) β γ	54.772 79.668 54.967 90.00 105.60 90.00	50.829 80.243 55.836 90.00 107.80 90.00	54.985 79.622 55.179 90.00 107.88 90.00
Resolution (Å) ^a^	20–2.18 (2.22–2.18)	20–1.93 (1.96–1.93)	20–2.33 (2.37–2.33)
R_merge_^b^ (%)	0.090 (0.548)	0.051 (0.247)	0.096 (0.488)
<I/σ>	20.8 (1.86)	31.2 (4.31)	20.1 (2.36)
Completeness (%)	100 (99.9)	100 (100)	100 (100)
Redundancy	5.4 (5.1)	4.7 (4.2)	5.6 (5.4)
**Refinement**	**P2_1_**	**P2_1_**	**P2_1_**
R_work_^c^/R_free_^d^ (%)	21.8/ 26.8	19.1/ 23.9	19.7/ 24.3
Unique reflections	23552	32119	19481
mean B factor (Å^2^)			
Protein	36.3	23.3	41.2
Ligand	34.5	27.7	37.6
Solvent	33.3	32.6	38.0
Ramachandran plot			
Most favored (%)	96.3	98.5	97.2
Add. allowed (%)	3.4	1.5	2.8
RMSD bond lengths (Å) bond angles (º)	0.004 0.086	0.005 1.183	0.006 1.291

^a^ Values in parentheses are for the highest resolution shell; ^b^ Rmerge = Σ|I-<I>|/ΣI, where I is the integrated intensity of a given reflection; ^c^. Rwork = Σ|F(obs) − F(calc)|/ΣF(obs); ^d^. Rfree = Σ|F(obs) − F(calc)|/ΣF(obs), calculated using 5% of the data.
